# Polyinosinic: Polycytidylic Acid and Murine Cytomegalovirus Modulate Expression of Murine IL-10 and IL-21 in White Adipose Tissue

**DOI:** 10.3390/v12050569

**Published:** 2020-05-22

**Authors:** Pablo Garcia-Valtanen, Ruth Marian Guzman-Genuino, John D. Hayball, Kerrilyn R. Diener

**Affiliations:** 1 Experimental Therapeutics Laboratory, UniSA Cancer Research Institute, Clinical and Health Sciences, University of South Australia, Adelaide 5000, Australia; ruth_marian.guzman@mymail.unisa.edu.au (R.M.G.-G.); john.hayball@unisa.edu.au (J.D.H.); 2 Robinson Research Institute and Adelaide Medical School, The University of Adelaide, Adelaide 5005, Australia

**Keywords:** Murine cytomegalovirus, polyinosinic: polycytidylic acid, white adipose tissue, regulatory B-cells, interleukin-10

## Abstract

White adipose tissue (WAT) produces interleukin-10 and other immune suppressors in response to pathogen-associated molecular patterns (PAMPs). It also homes a subset of B-cells specialized in the production of IL-10, referred to as regulatory B-cells. We investigated whether viral stimuli, polyinosinic: polycytidylic acid (poly(I:C)) or whole replicative murine cytomegalovirus (MCMV), could stimulate the expression of IL-10 in murine WAT using in vivo and ex vivo approaches. Our results showed that in vivo responses to systemic administration of poly(I:C) resulted in high levels of endogenously-produced IL-10 and IL-21 in WAT. In ex vivo WAT explants, a subset of B-cells increased their endogenous IL-10 expression in response to poly(I:C). Finally, MCMV replication in WAT explants resulted in decreased IL-10 levels, opposite to the effect seen with poly(I:C). Moreover, downregulation of IL-10 correlated with relatively lower number of Bregs. To our knowledge, this is the first report of IL-10 expression by WAT and WAT-associated B-cells in response to viral stimuli.

## 1. Introduction

White adipose tissue (WAT), long regarded as a tissue with only one purpose (storing energy), is now known to exert immune function [1,2] and regulate inflammation [3,4]. In concordance with its immune role, a heterogeneous population of innate and adaptive immune cells resides in WAT, including different subsets of macrophages, T-cells, natural killer (NK) cells and B-cells [5].

The role of WAT in the immune response to viral infections is of interest because this tissue is ubiquitously distributed and makes up a large percentage of the body weight of both the average person [6,7] and in vivo animal models such as the mouse [8]. Innate receptors involved in antiviral responses such as Toll-like receptor 3, a pattern recognition receptor (PRR) that recognizes viral dsRNA and its synthetic homologue polyinosinic:polycytidylic acid (poly(I:C)), are expressed in human adipocytes [9]. Interestingly, viruses such as the human immunodeficiency virus have developed mechanisms to survive within WAT when they are threatened, for example, by antiretroviral therapy [10]. Likewise, adenovirus infections have been consistently linked to obesity and the expansion of WAT [11].

Despite these recent advances, the immune role of WAT during viral infections is poorly understood. An interesting trait of WAT immune responses is that its endogenous cytokine production is skewed towards generating anti-inflammatory molecules such as interleukin-10 (IL-10) [12] or IL-1 receptor antagonist (IL-1Ra) [13]. Accordingly, WAT harbors a relatively abundant population of regulatory B-cells (Bregs), characterized by their ability to produce IL-10 [14]. This WAT anti-inflammatory biased immune function could be particularly important in the presence of viruses of the family *Herpesviridae*. The human cytomegalovirus (HCMV) and murine cytomegalovirus (MCMV) are both herpesviruses and have evolved to induce the expression of IL-10 in their respective hosts. Furthermore, HCMV encodes its own functional IL-10-like molecule [15]. Interleukin-10 is able to suppress the immune response enabling both HCMV and MCMV to establish life-long infections in their hosts [16,17,18,19,20].

In this study, we hypothesized that the ability of mouse WAT to produce IL-10 is strongly modulated in response to viral stimuli, and perhaps, cells specialized in production of IL-10 present in WAT could be involved. To test our hypothesis, we conducted in vivo and ex vivo experiments to measure production of IL-10 and other cytokines in gonadal white adipose tissue (GWAT), located next to the uterus and ovaries in female mice, and compared it to other organs. Our results show that, in comparison to spleen, liver, and uterus, GWAT depots in female C57Bl/6J mice produced large amounts of IL-10 and IL-21 in response to poly(I:C) in vivo via a TLR3-dependent pathway. In ex vivo GWAT explant cultures, IL-10-producing Bregs increased production of IL-10 in response to poly(I:C). In contrast, infection with MCMV down-regulated the expression of IL-10 and increased production of inflammatory cytokines IL-6 and TNFα. This change in IL-10 expression correlated with a reduction in the total number of B-cells, as well as the Breg subpopulation. To our knowledge, this is the first report that links viral pathogen-associated molecular patterns (PAMPs) and MCMV with the regulation of IL-10 in WAT, potentially via Bregs.

## 2. Materials and Methods

### 2.1. Animals

Female C57Bl/6J and TLR3 KO (6–8 weeks) mice were bred in house at the Reid Animal Facility, University of South Australia, and were housed in individually ventilated cages under standard specific-pathogen free conditions with food and water provided ad libitum. All animal experiments were carried out with dual approval from the University of South Australia and The University of Adelaide Animal Ethics Committees and conducted in accordance with National and Institutional ethical and regulatory guidelines.

### 2.2. Cells

Mouse fibroblast cell line 3T3-Swiss albino (ATCC, Manassas, VA, USA, CCL-92) was used to propagate and titrate murine cytomegalovirus. During the experiments 3T3-cells were passaged every three days and maintained in Dulbecco’s Modified Eagle’s Medium (DMEM, Sigma, Castle Hill, Australia, D5671) supplemented with penicillin streptomycin solution (GIBCO, Waltham MA, USA, 15140-122) 10 mM of HEPES (Sigma, H0887), 2 mM of L-Glutamine (GIBCO, 25030-081) and 10% of heat inactivated (56 °C for 30 min) fetal bovine serum (FBS; GIBCO, 26140-079).

### 2.3. Virus

The K181 strain of murine cytomegalovirus was propagated by infecting confluent monolayers of 3T3-cells in DMEM medium supplemented with 2% heat inactivated FBS. Three days post-infection (dpi) the media was collected and frozen, and fresh media was added to infected flasks. On day five after infection the media was collected and pooled with media from 3 dpi. The remaining adhered cells were scraped from the bottom of the flasks, resuspended in DMEM and sonicated (30 W twice for 5 s) to release intracellular mature MCMV virus particles. The sonicated homogenate and pooled infection media were then mixed and centrifuged (5000× *g* for 20 min at 4 °C) to remove cell debris. The clarified medium was then mixed with 2.3% *w*/*v* of NaCl and 5% *w*/*v* of Polyethylene glycol Mw 6000 (PEG; SIGMA, 81255) with constant agitation for one hour at 4 °C. The virus was then allowed to precipitate with PEG overnight at 4 °C. To collect the virus the medium was centrifuged at 15,000× *g* for 20 min at 4 °C and the PEG-virus pellets were resuspended in TES buffer (0.01 M Tris-HCI, 0.002 M EDTA and 0.15 M NaCI) at pH 7–7.2. Excess PEG was removed by centrifugation and the virus kept at −80 °C until used.

For virus titrations 2× MEM culture medium was prepared from MEM powder (Sigma M0268) as indicated by the manufacturer. Ninety percent confluent 3T3-cells in 24-well plates were infected with 100 µL of 10-fold serial dilutions (in complete DMEM with 2% FBS) of infected supernatants or tissue homogenates for 1 h at 37 °C rocking the plates intermittently. Then, the inocula were removed and a 1:1 mixture of 2× MEM with 4% FBS and a sterile 1% agar solution was added to the wells. Five days after infection a 10% buffered formalin solution was added to the wells and allowed to slowly diffuse and fix the infected cell monolayers over a 24 h period. The medium was then removed and cells were stained with crystal violet to facilitate virus plaque count.

### 2.4. In Vivo Poly(I:C) Injections and Organ Collection for Cytokine Evaluation

Female C57Bl/6J or TLR3 KO mice were injected with 20 mg/kg of poly(I:C) (Sigma, Castle Hill, Australia P1530). At 12 h after the injection all mice were exposed to isoflurane until unconscious. Then while still under the anesthetic the thoracic cage was open to expose the beating heart. A cut was performed in the right atrium and 15 mL of ice-cold PBS was injected slowly through the left ventricle to replace circulating blood with the saline solution. After perfusion all tissues were collected and placed in 1 mL of tissue lysis buffer (150 mM of NaCl, 50 mM of Tris-HCl at pH 8, 1% triton x100, and a freshly added protease inhibitor cocktail; Roche, Sydney, Australia 11873580001). All samples were minced with sterile scissors and incubated in the lysis buffer for 30–40 min with 10–20 s vortexing every ten minutes. Then, the resulting tissue homogenates were centrifuged at 14,000× *g* for 10 min and supernatants were collected and kept at −80 °C for protein analysis. Throughout the process all tissues were kept at 4–6 °C.

### 2.5. Adipose Tissue Explant Cultures and MCMV Infections

The estrous cycle phase (i.e., metestrus, diestrus, proestrus, or estrus) for each mouse was determined by observation of cell smears from vaginal lavages. Mice were euthanized with CO_2_ only if in the estrus phase. Immediately after, the mice abdomens were open in sterile conditions. The gonadal adipose tissue pads (GWAT), attached to the uterine neck and lining the uterine horns were excised and placed in complete DMEM culture medium at room temperature. All GWAT pads were thoroughly examined to ensure that they were free of lymph nodes. GWAT from each mouse was then cut into approximately 2 mm pieces and placed in sterile PBS with 5% FBS. Then PBS was poured through a cell strainer to collect the GWAT. This process was repeated three times to wash the tissue explants. Between four to six explants were placed in a single well in 6-well plates containing 4 mL of complete DMEM medium. The explant cultures were allowed to rest in the culture media for at least 24 h before any treatment or infection was performed on them.

For MCMV infections, explants were incubated for 90 min in the presence of 100,000 PFU/mL of MCMV (K181) or culture media (complete DMEM +10% ΔFBS, control) in a total volume of 4 mL well. During incubation the plates were gently rocked every 15 min to enhance virus contact with the explants. At the end of the incubation period media were removed and all wells were washed with 2 mL of DMEM +10% ΔFBS. Then 4 mL of fresh culture medium was added. At 1, 2, and 3 days post-infection 400 µL of the medium was collected from each well for cytokine evaluation and replaced with same amount of fresh medium.

### 2.6. Cytokine Evaluation

Different LEGENDplex^TM^ bead-array kits (BioLegend, San Diego, CA, USA) were used to detect mouse cytokines based on sandwich ELISA principles. Cytokine concentration was determined using standard curves obtained using recombinant cytokine standards provided in each kit. Bead signal readouts were obtained using the flow cytometer Becton DickinsonFACSAria™ Fusion and analyzed using LEGENDplex data analysis software.

### 2.7. Isolation of GWAT-Associated Stromal Vascular Fraction (SVF), Cell Staining, and Flow Cytometric Analysis

Cells of the SVF, containing GWAT-resident immune cells, were harvested from GWAT explants via enzymatic dissociation. To that end, fat pads were collected from the treatment wells and transferred into a 30 mm petri plate. After mincing the fat pads into smaller portions, the tissue was digested in 1 mL of the enzymatic mix composed of 900 μL of 2% FBS Roswell Park Memorial Institute (RPMI) cell culture medium, 100 μL of collagenase type I (10 mg/mL; Gibco Invitrogen, Carlsbad, CA, USA), and 1 μL of DNase I (4mg/mL; Roche, Basel, Switzerland). The petri dish was sealed and placed in an orbital shaker at 37 °C for 30 min with gentle rocking. Post digestion, the dissociated tissues were filtered through a 70 μm cell strainer to remove cell and tissue clumps then washed twice with PBS. The pelleted cells were then resuspended in 1 mL of Ammonium-Chloride-Potassium (ACK) lysis buffer (150 mM NH4Cl, 10 mM KHCO3, 0.1 mM Na2EDTA, pH 7.2) and incubated at room temperature for 5 min to eliminate red blood cells. Then, cells were washed once with PBS, counted, and dispensed at 1 × 10^6^ cells/well in a V-bottom 96-well plate (Corning Inc, NY, USA).

Multi-parameter flow cytometry was used to identify B- and T-cell populations in SVF and to measure the relative intracellular production of IL-10. The following panel of conjugated monoclonal antibodies (mAb) specific for the following markers were used: B220-BV650 Clone RA3-6B2 (BioLegend, San Diego, CA, USA), CD3e-APCCy7 Clone 145-2C11 (BD Biosciences, NJ, USA), IL-10-PE Clone JES5-16E3 (BioLegend), and Fixable Viability Dye eFluor™ 660 (eBioscience, San Diego, CA, USA). The cells were first incubated with anti-mouse CD16/CD32 (eBioscience) for Fc receptor blockade then stained with the cocktail of mAb in staining buffer (PBS with 2% FBS) for 40 min in the dark on ice. After staining, cells were fixed and permeabilized using the BD Cytofix/Cytoperm Kit (BD Biosciences). The permeabilized cells were then stained with IL-10-PE or the isotype for 30 min at room temperature. Cells were washed and transferred to FACS tubes for flow cytometry analysis. Unstained, single color, fluorescence minus one controls and isotype controls were used to set gates and identify positive populations. All data were analyzed using FlowJo software (Treestar, San Carlos, CA, USA).

### 2.8. Normalisation of Cytokine and Breg Data

In all in vivo and ex vivo experiments for spleen, liver, uterus, and GWAT tissue samples were weighed using precision laboratory-grade scales. Cytokine levels and cell count (from immune staining assays) readouts were then normalized against tissue weight measured at the analysis time point and expressed as pg of cytokine X/100 mg of tissue or cells/100 mg of tissue to allow comparison between experimental conditions and treatments.

### 2.9. Statistics

Statistical analysis and number of animals, experiments, or replicates are indicated for each experiment in their corresponding figure legends.

## 3. Results

### 3.1. In Vivo, Poly(I:C) Induces High Expression of Murine IL-10 and IL-21 in GWAT but not in Other Organs

**Figure 1:**
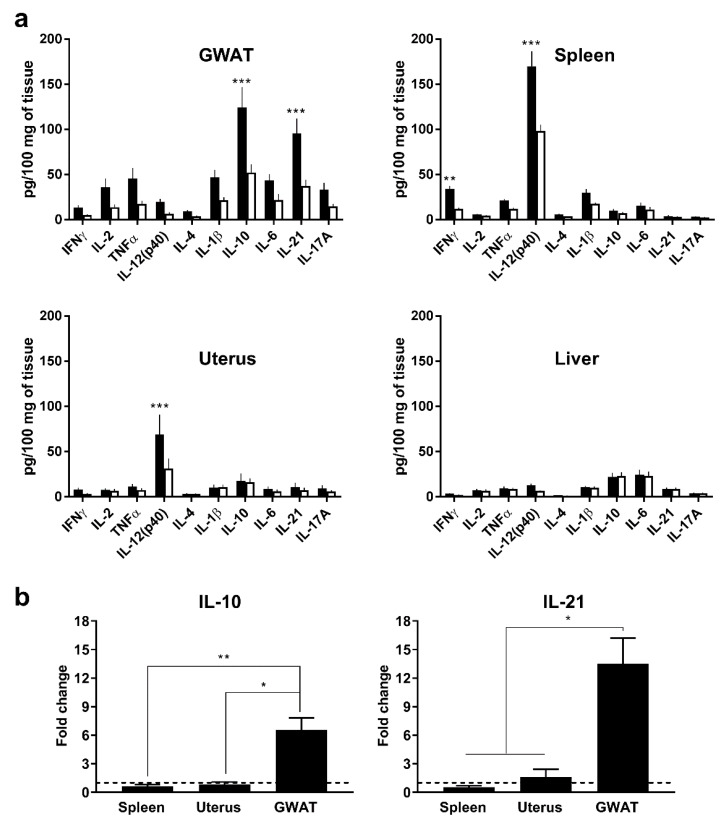
Organ-specific cytokine expression after polyinosinic:polycytidylic acid (poly[I:C]) stimulation. (**a**) Cytokine expression in C57Bl/6J (black columns) and TLR3 KO (white columns) mouse organs at 12 h after intraperitoneal administration of 20 mg/kg of poly(I:C). (**b**) Relative expression of IL-10 and IL-21 in C57Bl/6J mouse organs (expression was normalized by liver expression of same cytokines (value of 1) denoted with a dashed line). Bars represent average values from two independent experiments (total *n* = 7). Errors bars denote standard error of the mean (SEM). *, **, and *** denote *p* values ≤ 0.05, 0.01, and 0.001, respectively, according to Sidak’s multiple comparisons test.

To address our hypothesis, we assessed organ-specific cytokine responses to a systemic viral stimulus in vivo. Poly(I:C) was administered intraperitoneally to C57Bl/6J and TLR3 KO (as control) mice, and gonadal WAT (GWAT) pads from treated mice were collected along with other organs. Endogenous IL-10 expression and nine other cytokines were measured. Gonadal WAT pads were chosen as they reside adjacent to the uterus which can be exposed to sexually transmitted pathogens such as MCMV [21]. Furthermore, their relatively large size allows for accurate measurements of protein levels, and extraction of explants for ex vivo cultures. Results of cytokine analysis showed that, with the exception of interferon gamma (IFNγ) and IL-12(p40), the highest expression levels for all cytokines occurred in GWAT tissue from C57Bl/6J mice, compared to spleen, uterus, and liver (Figure 1a). Furthermore, GWAT IL-10 and IL-21 were significantly more expressed in C57Bl/6J (124.4 and 95.5 pg/100 mg of tissue, respectively) than in TLR3 KO mice (52.1 and 37.2 pg/100mg, respectively), indicative of a role for TLR3 signaling. In C57Bl/6J mice, liver, spleen, and uterus IL-10 expression was 22.1, 17.8, and 9.8 pg/100 mg of tissue, respectively, with no significant difference when compared to homologous samples in TLR3 KO mice. IL-12(p40) was most expressed in spleen and uterus of C57Bl/6J mice (169.8 and 69.1 pg/100 mg of tissue, respectively). Finally, expression data clearly showed that organ-wise, IL-10 and IL-21 were preferentially produced by GWAT tissue in response to poly(I:C), as shown in Figure 1b. Serum samples taken from C57Bl/6J mice contained relatively higher levels of IL12(p40) and IL-10 than their TLR3 KO counterparts, although this difference was not statistically significant (Figure S1).

### 3.2. GWAT Explants Stimulated Ex Vivo with Poly(I:C) Reproduce Endogenous IL-10 and IL-21 Responses Observed In Vivo

**Figure 2:**
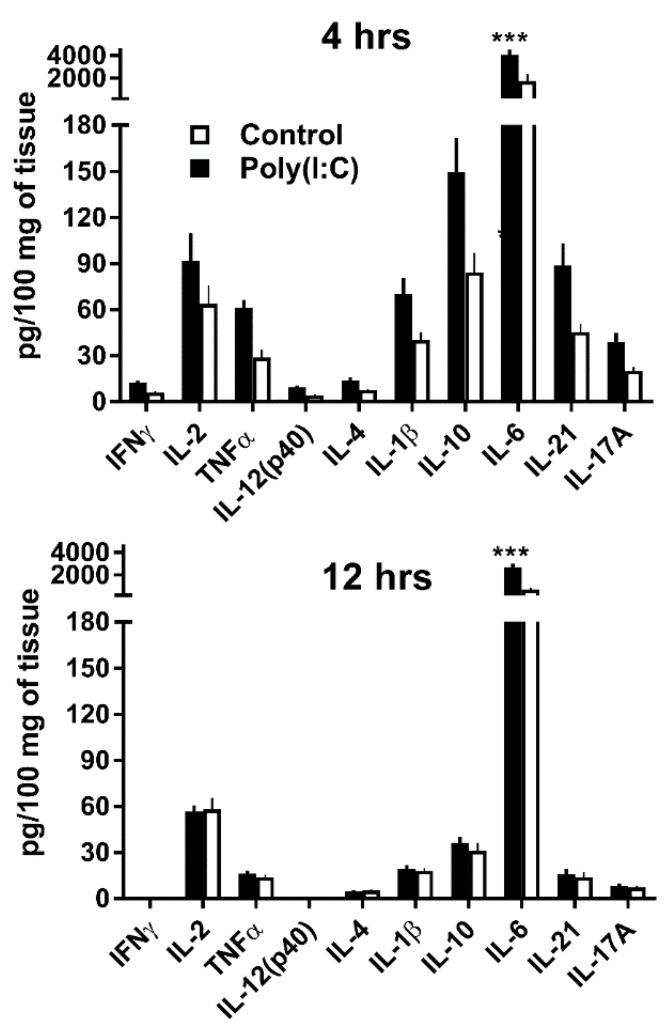
Endogenous cytokine expression in gonadal white adipose tissue (GWAT) explants in response to poly(I:C). GWAT explants were excised from C57Bl/6J mice and treated with 5 µg/mL of poly(I:C) or left untreated (black and white bars, respectively). Endogenous expression of cytokines were measured at 4 and 12 h post-treatment. Bars represent average values from two independent experiments (total *n* = 7). Errors bars denote SEM. *** *p* value ≤ 0.01 according to Sidak’s multiple comparisons test.

In order to facilitate our investigations and reduce the need for in vivo experiments we tested whether a GWAT explant ex vivo culture model could replicate the cytokine profiles observed for this organ in vivo. Because the cytokine expression kinetics of GWAT tissue in culture might differ from that of GWAT tissue in vivo, we tested two different time points after treatment: 4 and 12 h. Cultures of untreated GWAT explants from C57Bl/6J mice served as controls for cytokine expression. Expression at 4 h post-treatment was higher for all cytokines, compared to 12 h (Figure 2). As expected, IL-6, a pro-inflammatory cytokine known to be an early marker of poly(I:C)-stimulated immune responses [22,23,24], was expressed at ~4000 pg/100 mg of tissue at 4 h post-treatment and decreased significantly at 12 h (~2700 pg/100 mg of tissue). In vivo, different mechanisms and organs can contribute to a rapid clearing of IL-6 in mammalian species [25], therefore, higher IL-6 levels were expected in explant cultures. The expression profile of the rest of the cytokines in response to poly(I:C) was remarkably similar to our GWAT in vivo results. After IL-6, IL-10 was the most expressed cytokine as was in the in vivo results (149.5 pg/100 mg of tissue). IL-21 was also highly expressed in response to poly(I:C) reaching 88.5 pg/100 mg of tissue, similar to the in vivo results. A slight difference between the ex vivo and in vivo results was that IL-2 expression ex vivo was relatively higher in response to poly(I:C) at 91.6 pg/100 mg of tissue.

### 3.3. GWAT-Resident B-Cells Respond to Poly(I:C) by Increased Expression of IL-10

**Figure 3:**
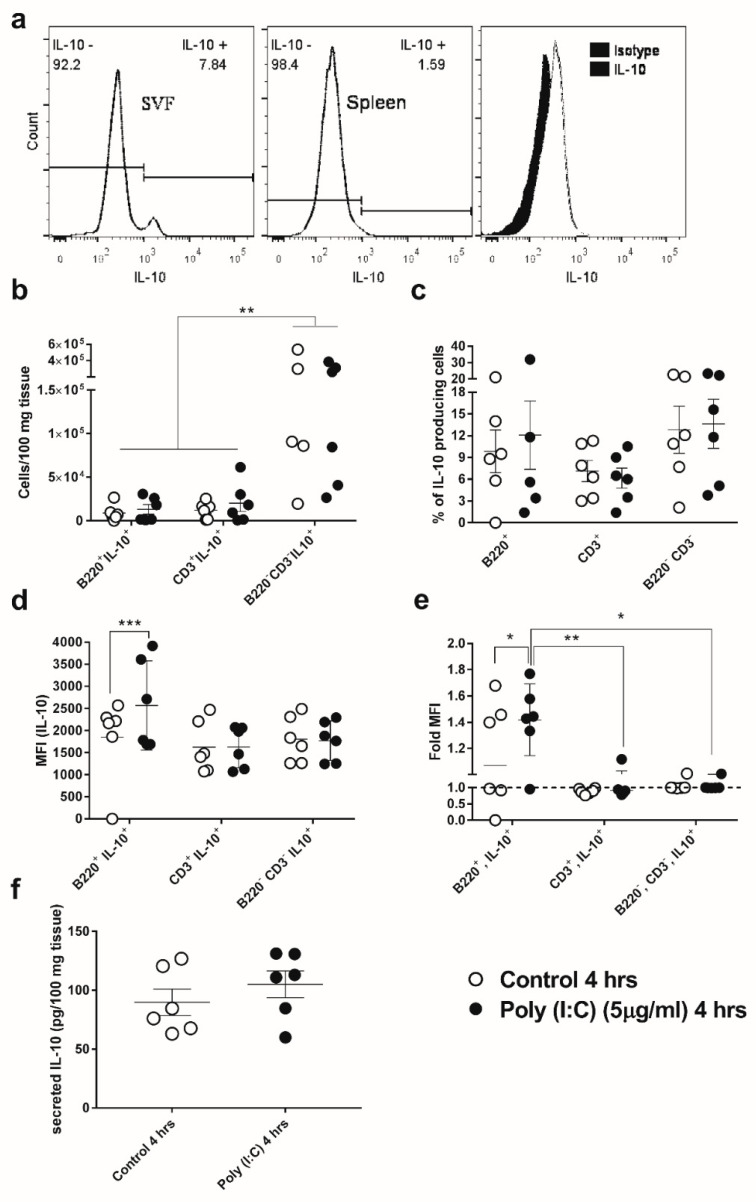
IL-10 expression in murine viable immune cells from the stromal vascular fraction (SVF) of GWAT explants after ex vivo exposure to 5 µg/mL of poly(I:C) for 4 h. (**a**) Murine B-cells (B220^+^) that express IL-10 are relatively more abundant in the GWAT SVF than in the splenocytes of the same mice. In these assays, samples were control-stained with isotype antibodies not specific to IL-10. Histograms are representative of the results obtained in all assays. (**b**) Total number of IL-10-expressing B-cells (B220^+^IL-10^+^), T-cells (CD3^+^IL-10^+^), and other cells in GWAT SVF (B220^−^CD3^−^IL-10^+^). (**c**) Percentage of total IL-10 expressing cells within each phenotype tested. (**d**) Level of IL-10 intracellular expression measured as the median fluorescence intensity (MFI) for B-cells, T-cells, and other cells. (**e**) MFI values in each group of cells was normalized against the average MFI for all cells (dashed line). (**f**) Tissue that was weight-normalized, secreted IL-10 in explant cultures treated or not treated with poly(I:C). Individual data points represent values obtained from explants from one individual mouse. Horizontal lines and error bars denote averages and SEMs, respectively. ***, ** and * denote *p* value ≤ 0.05, 0.01 and 0.001, respectively, according to Sidak’s multiple comparisons test.

Prior to this study, the existence of a relatively abundant population of IL-10-expressing B-cells, referred to as regulatory B-cells or Bregs, in the stromal vascular fraction (SVF) of WAT was reported by Nishimura et al. [14]. After establishing that IL-10 is produced by GWAT tissue in response to poly(I:C) in vivo and ex vivo, we investigated whether Bregs produced IL-10 in response to poly(I:C) in GWAT explants. In agreement with the literature [14], the percentage of resting B-cells that endogenously expressed IL-10 in GWAT SVF was higher than that in cultured splenocytes from the same mice (Figure 3a). When GWAT explants were stimulated ex vivo with poly(I:C), the number of cells that were identified as IL-10 expressing (IL-10^+^) did not change in the B-cell fraction, T-cell fraction, or for the non-B or T-cell fraction (B220^−^, CD3^−^; Figure 3b). While the total cell number differed between groups, the percentage of IL10^+^ cells was similar in all of the cell populations (Figure 3c). Although IL-10-expressing cell frequencies were not changed by ex vivo treatment with poly(I:C), when the median fluorescence intensity (MFI) was analyzed in each SVF cell fraction, we found that poly(I:C) induced upregulation of IL-10 in B-cells but not in the other cell phenotypes (Figure 3d). In addition, when MFI results were normalized by the average MFI for all SVF cells, we observed that, in the presence of poly(I:C), B-cells expressed on average 30–40% more IL-10 than the other cell phenotypes (Figure 3e), indicating that they respond to poly(I:C) by expressing more of this cytokine. Finally, analysis of the GWAT culture supernatants showed that IL-10 produced in the GWAT explants was secreted and released into the culture media, but differences between untreated and poly(I:C)-treated GWAT cultures were not statistically significant (Figure 3f).

### 3.4. MCMV Replicates in Gonadal White Adipose Tissue (GWAT)

**Figure 4:**
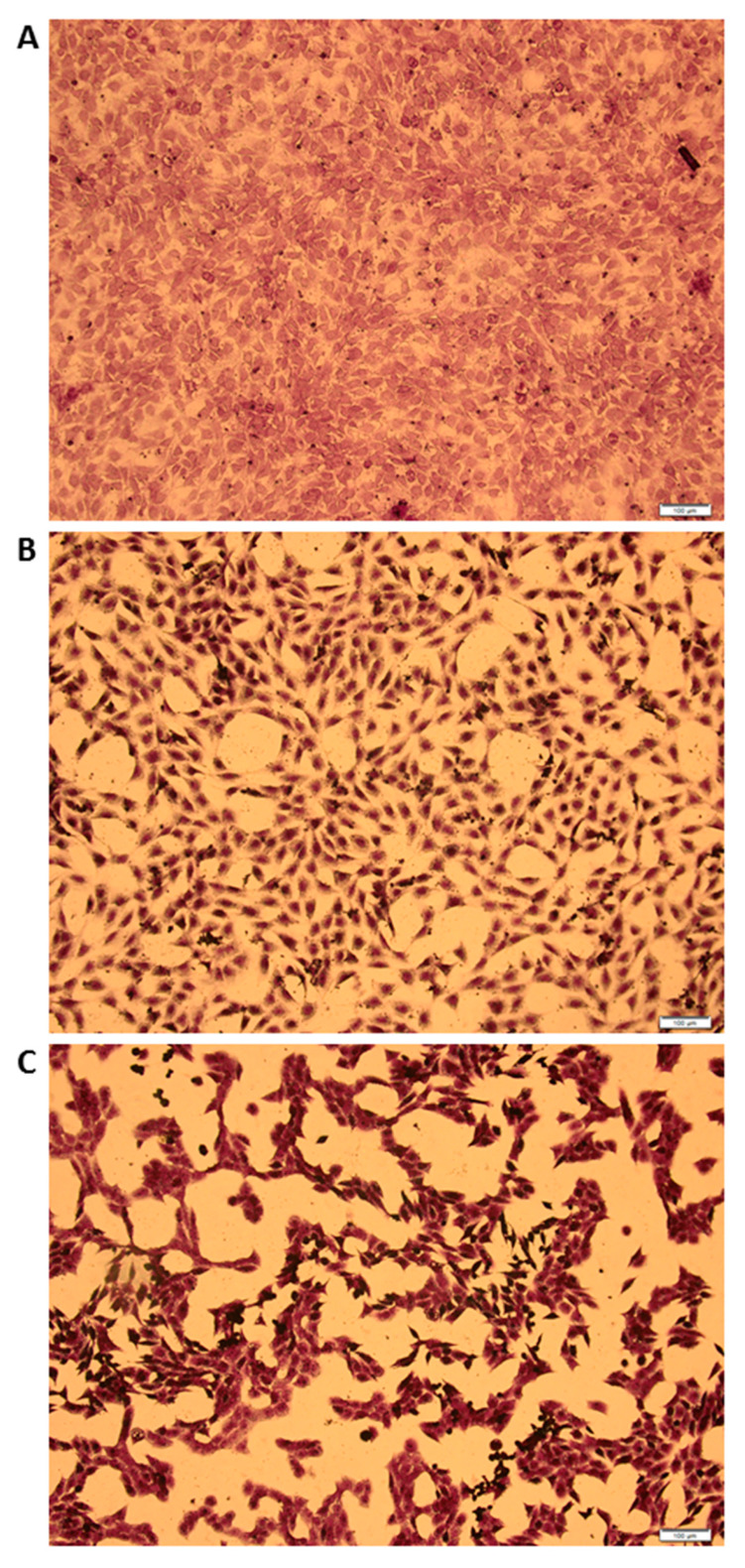
Murine Cytomegalovirus (MCMV) replicates in GWAT explant ex vivo cultures. GWAT explant cultures were infected with 100,000 PFU/mL of MCMV. Infection was allowed to progress for either 1 or 5 days. Control GWAT cultures were mock-infected (only cell culture medium) for 5 days. Supernatants from were collected from each of the cultures and used to infect 3T3-cells over a period of three days. Then, cells were stained with crystal violet. (**a**) Image of 3T3-cells infected with supernatants from mock-infected GWAT cultures, (**b**) 1-day MCMV-infected GWAT cultures or (**c**) 5-day MCMV-infected GWAT culture. Scale bar is 100 µm.

In order to test whether a true virus could modulate IL-10 expression in GWAT, we first investigated the ability of MCMV to replicate in this particular WAT depot. Murine Cytomegalovirus (CMV) replicated in GWAT explant cultures and was released into the supernatants as evidenced by the differential cytopathic effect of 3T3-cells infected with supernatants from MCMV-infected GWAT cultures 1 day post-infection (p.i.; Figure 4b) and 5 days p.i. (Figure 4c). As it is unlikely that MCMV completes a whole replication cycle in 24 h, cytopathic effect (CPE) seen in Figure 1b is likely to be the result of some residual virus from the inoculum after incubation with MCMV to initiate infection in GWAT cultures. However, the significant difference in CPE between day 1 and day 5 supernatants clearly indicates that MCMV replicated in GWAT explants. As expected, control 3T3-cells inoculated with supernatants from 5-day mock-infected GWAT cultures did not show CPE (Figure 4a). These results are in agreement with previous work that showed that mouse white [26] and brown adipose tissue [27] is susceptible to MCMV infection in vivo. In addition, it was recently shown that epidydimal white adipose tissue in male mice (equivalent to GWAT in female mice) is also infected by MCMV [28].

### 3.5. IL-10 Expression Is Down-regulated in MCMV Infected GWAT Explants

**Figure 5:**
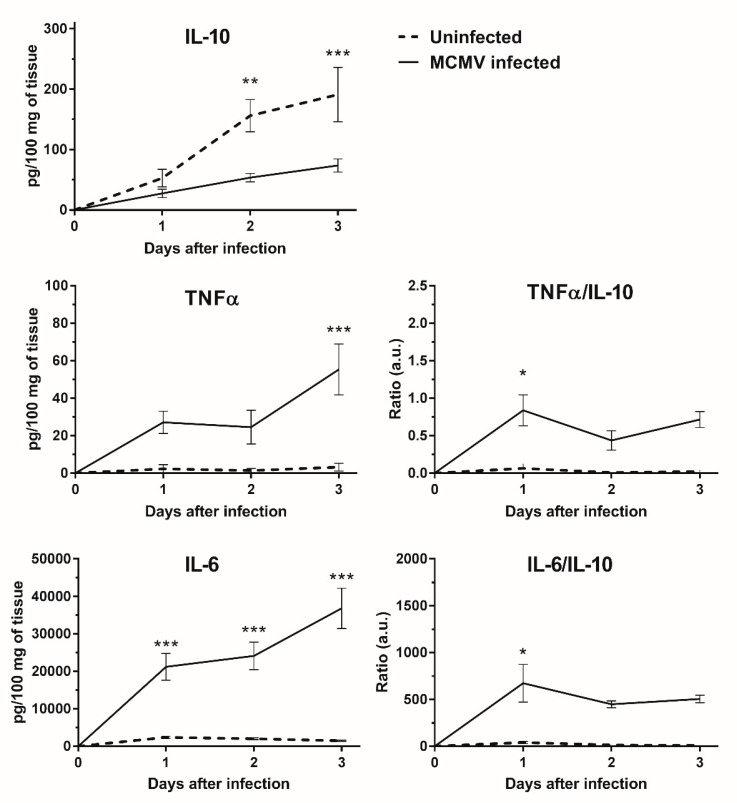
Inflammatory cytokines secreted by murine GWAT explant cultures after infection with MCMV. Explants were infected with 100,000 PFU/mL of MCMV or mock infected (isovolumetric TES buffer [0.01 M Tris-HCI, 0.002 M EDTA and 0.15 M NaCI at pH 7–7.2]) and the cytokine levels were evaluated in the supernatants daily for three days. Cytokine levels are normalized by the amount of tissue in each well at the start of infection. A total of six explants from different animals were tested in each condition in two independent experiments. Data points are average values (*n* = 6) from two different experiments. Error bars denote SEM. Ratios IL-6/IL-10 and TNFα/IL-10 are expressed in arbitrary units (a.u.). *, **, and *** correspond to *p* value ≤ 0.1, 0.05, and 0.01, respectively, according to Sidak’s multiple comparisons test.

Poly(I:C) is a useful and simple molecular tool to simulate innate viral responses in cells, organs, and in vivo systems that express any of the known cognate poly(I:C) receptors. Here, in a GWAT explant culture model, we established that GWAT endogenous expression of IL-10 is part of the early immune response to poly(I:C). Moreover, part of that IL-10 was released into the cell culture supernatant. In mice, MCMV relies on IL-10 expression upon infection to repress the mouse immune system and facilitate persistent infections in vivo [17]. Our results suggested that murine WAT could have an important role in producing and releasing IL-10 in vivo and therefore, facilitate MCMV infections. In order to assess this question in our ex vivo model, we measured IL-10 and other pro-inflammatory cytokines (IL-6 and TNFα) released in cell culture supernatants of uninfected and MCMV-infected GWAT cultures over a 3-day period (typical duration of MCMV replication cycle in vitro). Interleukin-10 was increasingly expressed and released in the supernatants of both uninfected and infected GWAT explants over the three days of culture (Figure 5). Surprisingly, IL-10 production was repressed in MCMV-infected explants compared to control uninfected cultures. On the contrary, IL-6 and TNFα were more expressed in infected supernatants, increasing concentration over the 3-day infection period. In uninfected cultures, IL-6 and TNFα showed significantly lower levels and no apparent trend over time. Interestingly, in infected cultures IL-6 and TNFα levels did not increase on day two while for IL-10 the upward trend was maintained at this time point and throughout the three days after infection with MCMV. This was more obvious when the ratios of IL-6/IL-10 and TNFα/IL-10 were calculated, both of which showed a pronounced drop in value on day two suggesting that IL-10, or perhaps other anti-inflammatory molecules not measured here, down-regulated TNFα and IL-6 in MCMV infected cultures.

### 3.6. Both the Number and Relative Percentage of Bregs Are Decreased in GWAT after MCMV Infection

**Figure 6:**
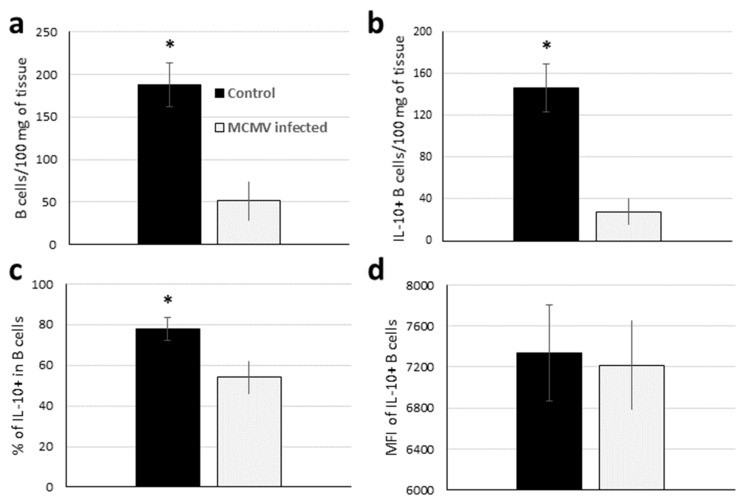
Regulatory B-cell number decreases in GWAT upon infection with MCMV. (**a**) Tissue weight-normalized number of viable B-cells (B220^+^ CD3e^−^) in the SVF from uninfected (black column) or MCMV-infected (grey) in 3-day GWAT cultures (as in Figure 5). (**b**) Tissue weight-normalized number of viable IL-10^+^ B-cells (Bregs) in the same tissues. (**c**) Percentage of IL-10 cells among viable B-cells. (**d**) Median fluorescence intensity associated with intracellular IL-10 production in IL-10^+^ B-cells. Columns and error bars denote average and standard deviation (*n* = 4). * *p* < 0.05 according to two tail *t*-test.

Because IL-10 was down-regulated upon MCMV infection, we quantified Bregs in the explants to see if a correlation between IL-10 production and Bregs could be established. The number of B-cells (all B-cells, including Bregs) was decreased 3.6-fold in MCMV-infected explants (Figure 6a). Cells that were not B- or T- cells (B220^−^, CD3e^−^) also decreased in number by 1.75-fold (data not shown). Upon infection, Bregs (IL-10^+^ B-cells) number decreased 5-fold, significantly more than that of all B-cells or other cells in SVF (Figure 6b). The percentage of Bregs within the B-cell population was, 22% lower in GWAT explants three days after infection with MCMV (Figure 6c), compared to uninfected controls. Here, increased IL-10 expression per cell was not observed, with the mean fluorescent intensity of IL-10 staining similar between infected explants and controls (Figure 6d). In addition, among non-B-cells (the majority of cells in SVF), the number of IL-10-producing cells was 146 and 15 on average for uninfected and infected GWAT in the same explants, numbers similar to those found in B-cells and indicative of the relatively important role of Bregs in regulating/producing IL-10 in this tissue.

## 4. Discussion

Adipose tissue is one of the most abundant tissues in humans, the majority of it is WAT. The average body of an American man and woman contains ~28% and 40% of adipose tissue, respectively, [6] and in laboratory mouse strains the percentage of WAT ranges from ~15% to 40% [8]. Yet, despite its abundance and being ubiquitously distributed, WAT has not received as much attention as other organs when studying immune responses to pathogens such as viruses. We observed that poly(I:C) evoked strong, TLR3-dependent cytokine responses in GWAT depots both in vivo and ex vivo. Others have shown that murine pre-adipocytes and adipocytes mount antiviral-like, pro-inflammatory responses to poly(I:C) [29] in concordance with our in vivo results. Although other intracellular innate immune cytosolic receptors such as melanoma differentiation-associated 5 (MDA5) and retinoic acid-induced 1 (RIG-1) expressed in WAT cells can participate in sensing poly(I:C) [29], in vivo systemic exposure to poly(I:C) is more likely to be sensed by TLR3, expressed on the outer cell membrane, as evidenced by the in vivo cytokine expression in TLR3 KO mice.

In vivo, the response of GWAT to poly(I:C) resulted in high expression of IL-21 and IL-10. Interleukin 21 negatively regulates regulatory T-cells in GWAT in male mice favoring chronic inflammation in obese mice [30]. Investigating the role of IL-21 in modulating regulatory T-cells or other immune cell subsets in WAT and its overall effect on viral replication could be worthwhile in future studies. White adipose tissue responses to bacterial PAMPs have been shown to be skewed towards an anti-inflammatory response characterized by expression of IL-10 [12] and IL-1Ra [13,31] in similarity to our in vivo results for IL-10 upon exposure to poly(I:C). A closer analysis of the cells within the SVF of GWAT explants revealed that a subset of B-cells responded to poly(I:C) increasing intracellular expression of IL-10. In mice, WAT depots are relatively rich in regulatory B-cells that express IL-10 which play an important role in regulating inflammation in obesity [14]. Others have found that IL-10-expressing cells localized in the peritoneum can be critical for pregnancy success in female mice [32]. We recently showed that uterine Bregs number are increased in the embryo implantation period in the mouse downregulating potentially harmful T-cells subsets [33]. Whether Bregs in GWAT depots (in close contact with the uterus) could have a role in pregnancy controlling inflammation during viral infections is worth investigating.

In humans, CMV causes intrauterine infections associated with life-long, severe sequelae in 0.2–2.6% of all babies [34,35]. Part of the current understanding of MCMV and HCMV biology is that IL-10 plays a role in the establishment of life-long persistent infections in their respective hosts [16,17]. Surprisingly, in our explant model MCMV down-regulated IL-10 expression and secretion in GWAT explants while increasing pro-inflammatory cytokines (TNFα and IL-6). Interleukin-10 expression peaked three days post-infection in vitro, while others have reported that in vivo, murine serum IL-10 peaked on day five post-infection during MCMV infections [17]. Murine CMV was able to infect GWAT explants in culture. In vivo, MCMV can infect different depots of both white [26,28] and brown [27] adipose tissue depending on the route of infection, including epidydimal adipose tissue, the male homologue of GWAT in female mice [28]. B-cell number was drastically reduced in MCMV-infected GWAT cultures. While, partly, MCMV could cause cell death during the course of its replicative cycle, the number of Bregs (IL-10 producing B-cells) was reduced further due to infection. Whether MCMV could preferentially infect these cells or whether it simply down-regulates the expression of IL-10 was not determined in this study and it deserves special attention in the future. In MCMV infected white adipose tissue, MCMV-specific CD8 T-cells can infiltrate the tissue and cause inflammation measurable long after infection as recently shown by Contreras et al. [28]. In their study, the specific role of B-cells within the SVF was not assessed, but their data, similar to ours, suggests that MCMV benefits from debilitating the anti-inflammatory role of WAT.

In summary, this study highlights that WAT and in particular GWAT depots are an abundant source of IL-10 and IL-21 in the context of a viral infection. Moreover, WAT-resident B-cells are likely to play an important role regulating how much IL-10 is produced when exposed to viral PAMPs or whole virus particles. Murine CMV may have repressed the ability of GWAT to produce and release IL-10, highlighting the importance of future studies to investigate the interaction between WAT depots and other tissues during viral infections with MCMV and other viruses.

## Supplementary Materials

The following are available online at https://www.mdpi.com/1999-4915/12/5/569/s1.
