# Supplementary material for: Polyinosinic: Polycytidylic Acid and Murine Cytomegalovirus Modulate Expression of Murine IL-10 and IL-21 in White Adipose Tissue
Source: Viruses. 2020 May 22;12(5):569. doi: 10.3390/v12050569 (PMC7290755; doi:10.3390/v12050569)
Supplement: Supplementary file 1 [file viruses-12-00569-s001.pdf]

Polyinosinic:polycytidylic acid and murine cytomegalovirus strongly modulate expression of murine IL-10 and IL-21 in white adipose tissue.

Pablo Garcia-Valtanen , Ruth Marian Guzman-Genuino , John D. Hayball and Kerrilyn R. Diener.

Supplementary material

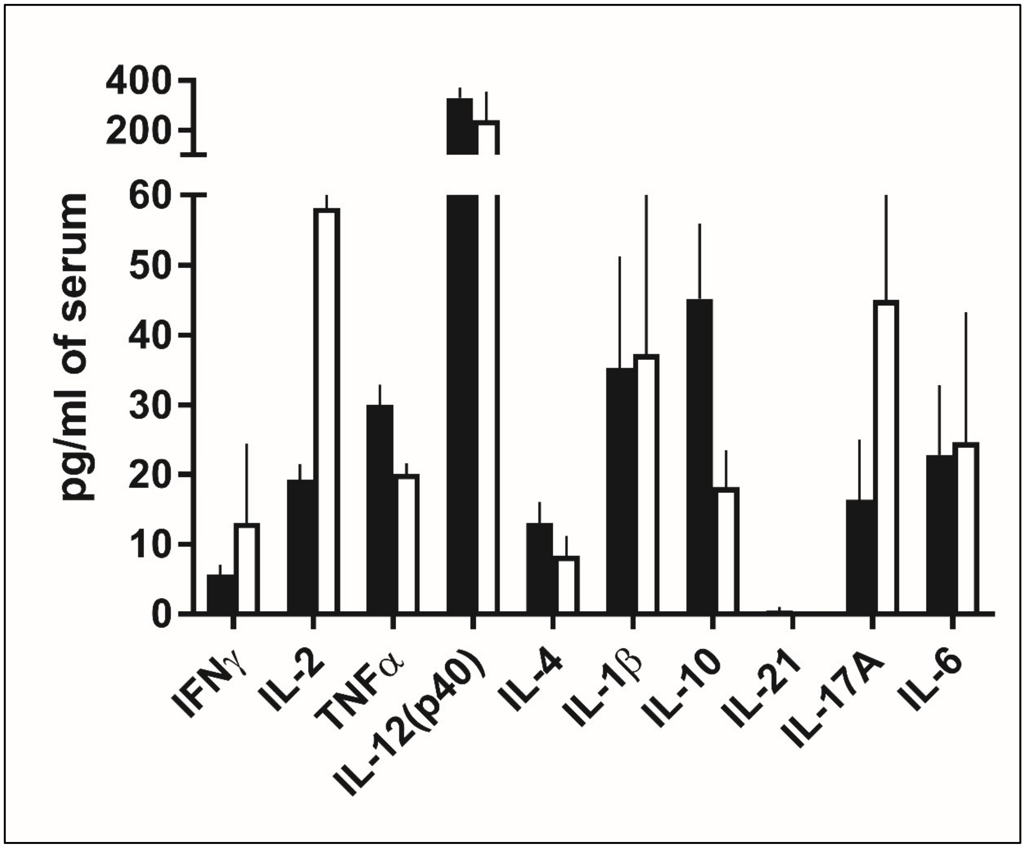

Figure S1. Serum cytokine expression in C57Bl/6J (blank bars) and TLR3 KO (white bars) mice 12 hours after intraperitoneal administration of 20 mg/kg of poly(I:C). Bars represent average values from two independent experiments (n=7). Errors bars denote SEM. No statistically significant differences were found according to Sidak's multiple comparisons test.
